# A Decentralized Kidney Transplant Biopsy Classifier for Transplant Rejection Developed Using Genes of the Banff-Human Organ Transplant Panel

**DOI:** 10.3389/fimmu.2022.841519

**Published:** 2022-05-10

**Authors:** Myrthe van Baardwijk, Iacopo Cristoferi, Jie Ju, Hilal Varol, Robert C. Minnee, Marlies E. J. Reinders, Yunlei Li, Andrew P. Stubbs, Marian C. Clahsen-van Groningen

**Affiliations:** ^1^ Department of Pathology and Clinical Bioinformatics, Erasmus MC, University Medical Center Rotterdam, Rotterdam, Netherlands; ^2^ Division of HPB and Transplant Surgery, Department of Surgery, Erasmus MC, University Medical Center Rotterdam, Rotterdam, Netherlands; ^3^ Erasmus MC Transplant Institute, Erasmus MC, University Medical Center Rotterdam, Rotterdam, Netherlands; ^4^ Companion Diagnostics and Personalised Healthcare, Omnigen BV, Delft, Netherlands; ^5^ Department of Internal Medicine, Erasmus MC, University Medical Center Rotterdam, Rotterdam, Netherlands; ^6^ Institute of Experimental Medicine and Systems Biology, Rheinish-Westphalian Technical University Aachen University (RWTH), Aachen, Germany

**Keywords:** kidney transplantation, gene expression, graft rejection, diagnosis, pathology, transcriptomics, bioinformatics, machine learning

## Abstract

**Introduction:**

A decentralized and multi-platform-compatible molecular diagnostic tool for kidney transplant biopsies could improve the dissemination and exploitation of this technology, increasing its clinical impact. As a first step towards this molecular diagnostic tool, we developed and validated a classifier using the genes of the Banff-Human Organ Transplant (B-HOT) panel extracted from a historical Molecular Microscope^®^ Diagnostic system microarray dataset. Furthermore, we evaluated the discriminative power of the B-HOT panel in a clinical scenario.

**Materials and Methods:**

Gene expression data from 1,181 kidney transplant biopsies were used as training data for three random forest models to predict kidney transplant biopsy Banff categories, including non-rejection (NR), antibody-mediated rejection (ABMR), and T-cell-mediated rejection (TCMR). Performance was evaluated using nested cross-validation. The three models used different sets of input features: the first model (B-HOT Model) was trained on only the genes included in the B-HOT panel, the second model (Feature Selection Model) was based on sequential forward feature selection from all available genes, and the third model (B-HOT+ Model) was based on the combination of the two models, i.e. B-HOT panel genes plus highly predictive genes from the sequential forward feature selection. After performance assessment on cross-validation, the best-performing model was validated on an external independent dataset based on a different microarray version.

**Results:**

The best performances were achieved by the B-HOT+ Model, a multilabel random forest model trained on B-HOT panel genes with the addition of the 6 most predictive genes of the Feature Selection Model (*ST7*, *KLRC4-KLRK1*, *TRBC1*, *TRBV6-5*, *TRBV19*, and *ZFX*), with a mean accuracy of 92.1% during cross-validation. On the validation set, the same model achieved Area Under the ROC Curve (AUC) of 0.965 and 0.982 for NR and ABMR respectively.

**Discussion:**

This kidney transplant biopsy classifier is one step closer to the development of a decentralized kidney transplant biopsy classifier that is effective on data derived from different gene expression platforms. The B-HOT panel proved to be a reliable highly-predictive panel for kidney transplant rejection classification. Furthermore, we propose to include the aforementioned 6 genes in the B-HOT panel for further optimization of this commercially available panel.

## Introduction

A reliable diagnostic system for rejection is needed for an optimal therapeutic approach of kidney transplant recipients (1). Currently, kidney transplant rejection is commonly classified following the Banff consensus criteria, a classification that relies on the evaluation of canonical traits (i-, t-, and v-lesions for T-cell-mediated rejection (TCMR); ptc-, g-, and cg-lesions, staining for C4d, and circulating donor-specific antibody for antibody-mediated rejection (ABMR)) (2). A grading system to evaluate continuous biological processes should be reproducible by one observer (with low intraobserver error) and between observers (with low interobserver error) (3). However, high interobserver disagreement (Cohen’s kappa coefficient ranging between 0.2 and 0.4) characterizes the histological diagnosis and classification of biopsies obtained from patients suspected to be undergoing graft rejection using the Banff criteria (4).

Over the past years, multiple study groups focused on the development of more reliable diagnostic systems such as molecular systems for allograft pathology. A centralized rejection diagnostic system called “The Molecular Microscope^®^ Diagnostic System” (MMDx) was developed based on the microarray assessment of messenger RNA levels performed over post-transplant kidney biopsies and their relationship with histologically determined clinical phenotypes (5). The abovementioned system estimates the probability that a sample has features of TCMR, ABMR, any type of rejection, tubular atrophy/interstitial fibrosis, or progression to failure. The same system also provides clinically valuable predictions for samples with difficult histological diagnoses. However, even though this centralized microarray-limited approach could minimize the impact of variation in measurements between laboratories (6), it limits in the meantime the availability and the impact of a diagnostic tool for other centers.

A decentralized, open-access system to diagnose rejection and classify TCMR and ABMR that is compatible with different gene expression assessment platforms would be crucial for optimal dissemination and exploitation of this technology. In an attempt to move the research community towards a decentralized diagnostic system, the Banff Molecular Diagnostics Working Group (MDWG), in association with the industry partner NanoString^®^, developed a non-proprietary 770 genes panel called the Banff-Human Organ Transplant (B-HOT) Panel. The B-HOT panel includes the most relevant genes for what concerns transplant rejection, tolerance, viral infections, and innate and adaptive immune response according to peer-reviewed literature (7). The development of a classifier that is based on a smaller and standardized subset of genes that can be measured with different techniques could be the first step towards the development of a decentralized and multiplatform-compatible kidney transplant biopsy classifier.

The present study aims at developing a decentralized molecular kidney transplant biopsy classifier to diagnose transplant rejection and ultimately improve and fine-tune this classification. Achieving a more precise diagnosis will aid transplant clinicians in the quick elaboration of a tailored therapeutic plan. In this study, a classifier based on the random forest algorithm was developed using microarray data from an online public dataset (8). The available data was filtered to contain only those probes included in the B-HOT Panel. Moreover, we compared the discriminating power of the B-HOT panel to that of sequential forward feature selection applied to the whole microarray gene set in order to assess its performance in a clinical scenario. Successively, the system was validated on another publicly available dataset based on a different microarray version (9).

## Materials and Methods

All code presented in this section is available in the following GitHub repository: https://github.com/ErasmusMC-Bioinformatics/KidneyRejectionClassifier.

### Data Collection and Preprocessing

**Figure 1:**
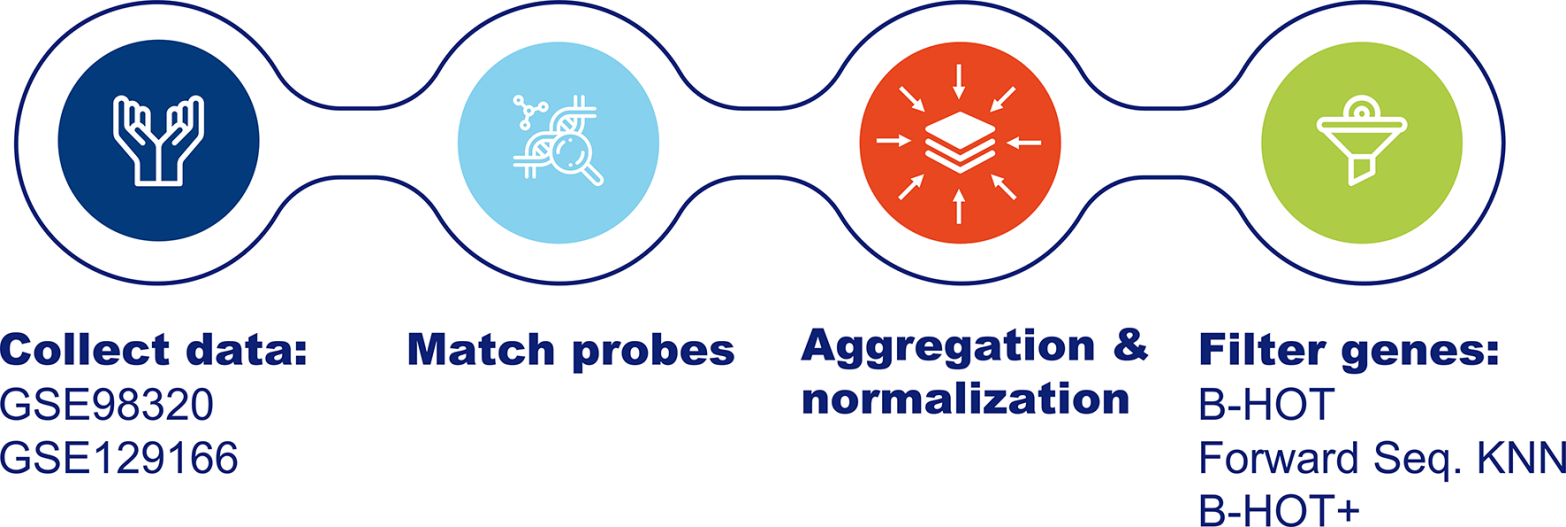
Overview of Data Collection and Preprocessing. Data has been retrieved from the GEO dataset repository. Probes have been matched using raw annotation files and then aggregated based on the median values and using *robustscale* per feature. Finally, the genes from the Banff Human Organ Transplant B-HOT panel have been filtered. B-HOT, Banff-Human Organ Transplant; GEO, Gene Express Omnibus; KNN, K-nearest neighbors.

An overview of data collection and preprocessing is presented in **Figure 1**. Two public gene expression datasets (GSE98320 and GSE129166) from the Gene Expression Omnibus database were collected as raw data matrices to serve as training and independent validation datasets respectively. A summary of the composition of both sets is shown in **Table 1**. A summary of the demographics and the clinical characteristics of the GSE datasets (GSE98320 and GSE129166) is shown in **Table 2** (8, 9). As previously reported (8), GSE98320 was obtained by running 1,208 biopsy samples from 1,045 patients at 13 international centers on Affymetrix hgu219 PrimeView microarray chips. Diagnoses derived from the annotations of the dataset GSE98320 were MMDx archetypes, specifically three types of ABMR (early-stage, fully-developed, and late-stage), TCMR, NR, and mixed rejection. The cohort is composed of 774 biopsies classified as NR and 434 biopsies classified as rejection (275 as ABMR, 51 as late-ABMR, 27 as Mixed, and 81 as TCMR). As previously described (9), the GSE129166 was obtained running 117 peripheral blood samples and 95 kidney biopsy samples on Affymetrix GeneChip Human Genome U133 Plus 2.0 microarray chips. Diagnoses derived from the annotations of the dataset GSE129166 were histologically assessed categorical classes, specifically ABMR, TCMR, NR, mixed rejection, and borderline rejection. After discarding the peripheral blood samples, the resulting cohort was composed of 60 biopsies classified as NR and 35 biopsies classified as rejection (15 as ABMR, 2 as TCMR, 2 as Mixed, and 16 as Borderline). The main clinical interest in the use of this classifier is to distinguish between rejection and NR and subsequently distinguishing the presence of ABMR as this is not always clearcut when evaluating the biopsy. For this reason and to make the predicted categories homogeneous between the two datasets, ABMR and late-ABMR samples from GSE98320 have been grouped under the ABMR category and all the samples classified as Mixed or Borderline have been removed. For the same reason GSE129166 was considered a valid dataset for validation. The limitation concerning the scarcity of TCMR samples is addressed in the Discussion section. After samples labeled as Mixed and Borderline were removed, 1,181 and 77 samples were left in the training set and in the validation set respectively.

**Table 1:** Overview of datasets composition.

Dataset	NR	ABMR	TCMR
**GSE98320**	774	326	81
**GSE129166**	60	15	2

ABMR, Antibody-Mediated Rejection; NR, Non-Rejection; TCMR, T-Cell-Mediated Rejection.

**Table 2:** Demographics and clinical characteristics GSE data sets.

Patient Demographics		GSE98320 (*n* = 1,045)	GSE129166 (*n* = 365)
Mean Recipient Age (range)		52 (18-86)	50.2 (2.7-78.5)
Recipient sex (male/female)		559/486 (53%/47%)	224/141 (61.4%/38.6%)
Ethnicity	European	522 (50%)	318 (87.8%)
	African	83 (8%)	6 (1.7%)
	Other/Not available	440 (42%)	38 (10.5%)
Mean donor age (range)		43 (0.03-85)	50.6 (5.0-91.0)
Donor sex		512/533 (49%/51%)	177/180 (50.4%/49.6%)
Donor type (deceased/living)		692/353 (66%/44%)	278/83 (77.0%-23.0%)
**Clinical characteristics at the time of biopsy**		**GSE98320 (*n* = 1,208)**	**GSE129166 (*n* = 387)**
Median time of biopsy after transplant in days (range)		591 (1-11,453)	908 (6-12,564)
Early biopsies (<1 year)		507 (42%)	207 (53.5%)
Late biopsies (>= 1 year)		701 (58%)	180 (46.5%)

Adapted from (8, 9).

Preprocessing of expression datasets was done using R version 4.1 (10). The probes of the different microarrays were mapped to Entrez IDs as stable gene identifiers using the annotation files provided by the manufacturer. When multiple probes were annotated to be the same gene, the gene expression level was aggregated based on their median value to produce expression values less sensitive to outlier transcripts. Genes not measured on both microarray versions or with ambiguous mapping (e.g. assigned to multiple genes) were removed from the dataset. The *ComBat* package was used for adjusting possible batch effects introduced by the difference between the two microarray platforms (9). Afterward, the gene expression values of the two datasets were scaled separately using the *robustscale* function from the *quantable* package (11). This method removes the median and scales according to the quantile range, thereby removing variance introduced by outliers.

### Principal Component Analysis

Principal component analysis was executed before and after the application of the above-described preprocessing steps using the *PCAtools* package in R (12). Biplots of the 1^st^ and 2^nd^ principal components were analyzed to evaluate the distinction of the different patient groups, as well as to compare the variation in expression introduced by differences in gene expression measurement techniques. The biplots before and after transformation were compared to determine if the batch effect removal was successful.

### Rejection Classifier Development

**Figure 2:**
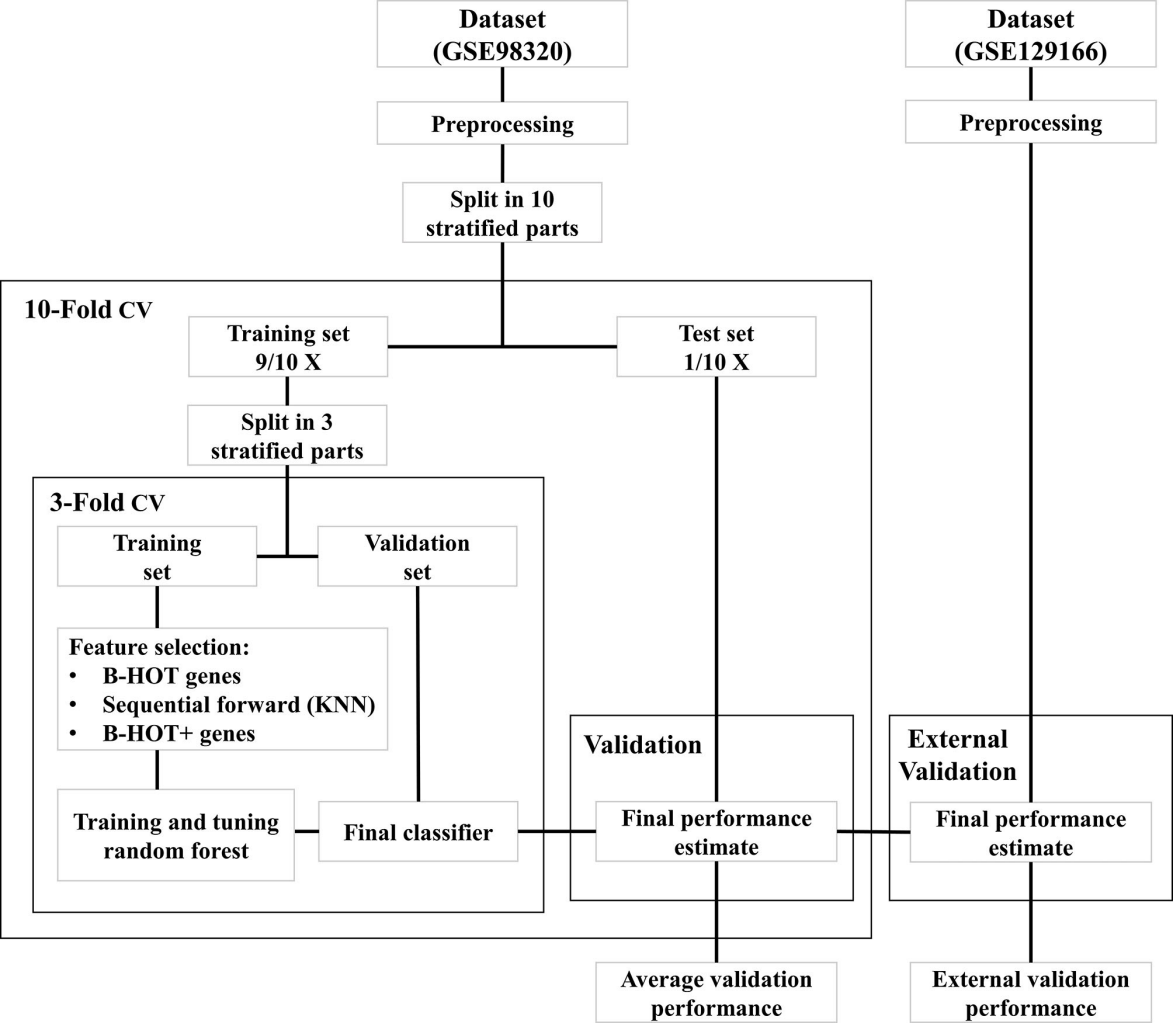
Overview of model development workflow. B-HOT, Banff-Human Organ Transplant; CV, Cross-Validation; KNN, K-nearest neighbors.

All modeling was executed using Python 3.9 (13) with the *scikit-learn* module (14). An overview of the model development strategy is presented in **Figure 2**. For each of the hereafter mentioned models, a nested cross-validation (CV) procedure using ten outer folds and three inner folds was implemented. The folds were stratified for the different classes and the same split was used for each of the developed models. The selection of parameters and features was executed within the inner CV folds to prevent overfitting the training dataset. Based on the outer CV prediction probabilities for the different classes, the overall accuracy was calculated, as well as the precision, recall, Area Under the ROC Curve (AUC), and F1 scores for each of the three classes. The AUC scores were determined for the prediction of each class against all others.

#### B-HOT Model

The B-HOT panel was selected as a suitable feature set for rejection classification. The annotation file from NanoString^®^ was subjected to manual curation which included removal of viral genes undetectable by microarray and changing ambiguous nomenclature. Gene expression data was filtered after the data collection and preprocessing steps. A first random forest model was trained on the B-HOT panel genes within the nested cross-validation scheme. Parameters of the model were tuned within the inner loops using a grid search algorithm. Tested classification parameters of the model are available on GitHub. Based on the overall cross-validation accuracy of the models trained on the outer folds, the best model was selected and fitted to the entire training set. This model was identified as the B-HOT Model.

#### Feature Selection Model

To test the validity of the B-HOT panel feature set, a second random forest model was trained using a wrapper feature selection technique on the complete set of genes measured by both microarray versions. For this purpose, a sequential forward feature selector was implemented that used a k-nearest neighbors classifier to sequentially select the best features. K was set to three to limit the computational load and the number of features to select was limited to a hundred to match the maximum number of features selected by the random forest model. The feature selection was implemented within the inner CV loop and the random forest model was then trained with the same classification parameters as the B-HOT model. This model was identified as the Feature Selection Model.

#### B-HOT+ Model

To test potential new candidate genes for the B-HOT panel, a third model was trained by sequentially adding the most important features from the Feature Selection Model to the B-HOT genes until the average cross-validation accuracy could not be improved. The most important Feature Selection Model features were defined as those with the highest mean Gini impurity decrease that is built-in within the *scikit-learn* module. This third model was developed using the same classification parameters as before and identified as the B-HOT+ Model. The cross-validation metrics of the three strategies were compared to determine the most suitable feature set. Based on the average accuracy of the cross-validation folds, the best-performing model was selected and fitted to the complete training dataset.

### Measuring Performance Using Independent Micro-Array Data

Out of the three developed models, only the best-performing model was tested on the independent validation set GSE129166 to determine the validity of the model independently from the training data and microarray version. Predictions of ABMR, TCMR, and NR classes were made for the samples of the GSE129166 dataset. Based on the prediction probabilities for the different classes, the overall accuracy was calculated, as well as the precision, recall, AUC, and F1 scores for the ABMR and NR classes. The AUC scores were determined for each class against all others.

## Results

### Preprocessing

**Figure 3:**
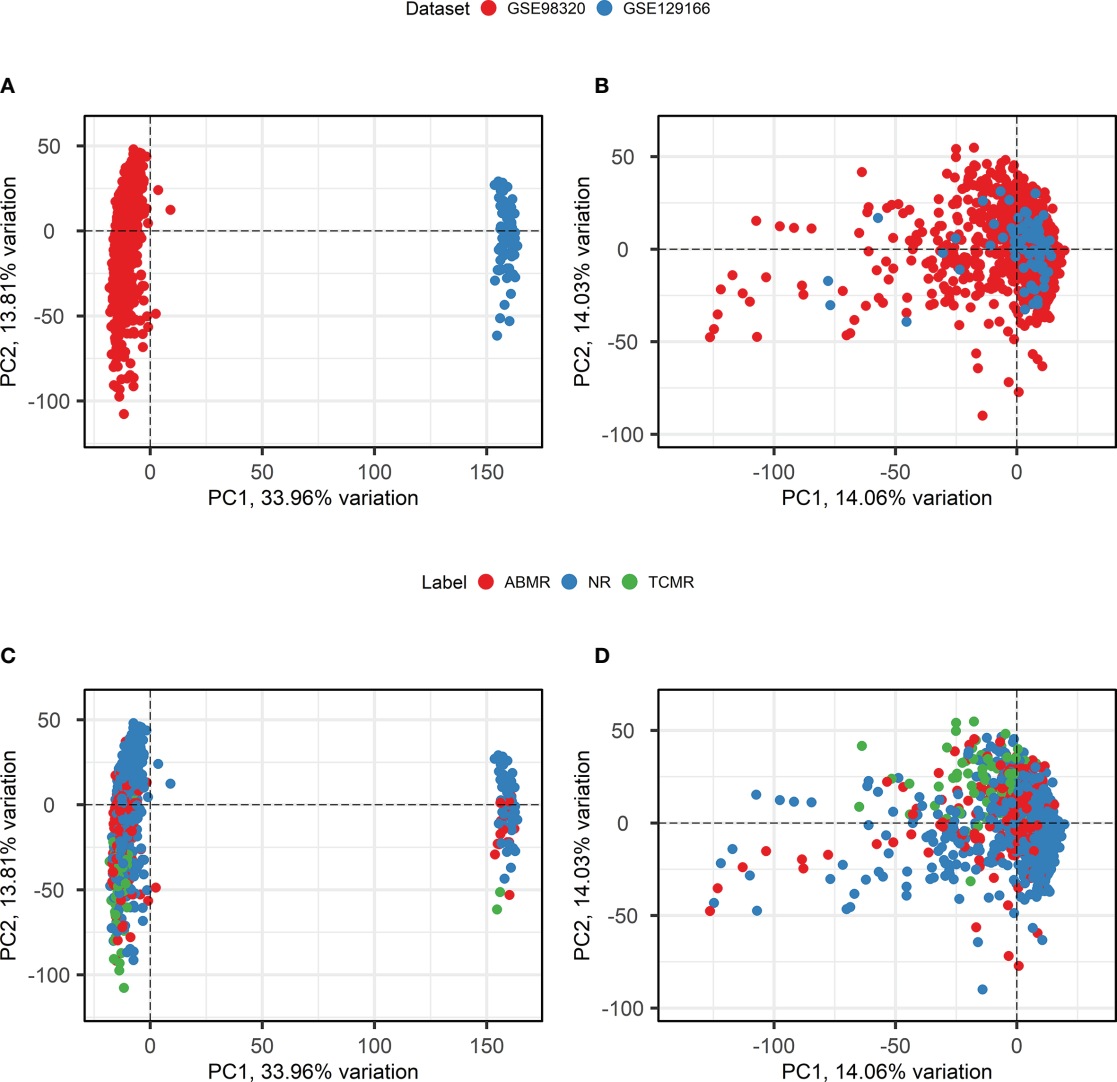
Principal component analysis and Batch Correction. **(A, B)**. Principal component analysis biplots of samples labeled based on their origin dataset before **(A)** and after **(B)** batch effect removal using *ComBat*. **(C, D)**. Principal component analysis biplots of samples labeled based on their diagnosis before **(C)** and after **(D)** batch effect removal using *ComBat*. ABMR, Antibody-Mediated Rejection; NR, Non-Rejection; PC, Principal Component; TCMR, T-Cell-Mediated Rejection.

Batch effect correction is necessary when combining datasets measured using different microarray platforms. To apply *ComBat* batch effect correction, only genes that are present in both the involved datasets must be selected. After probe matching and aggregation on the gene level, 18,945 genes overlapped between the two datasets. An overview of principal component analysis and batch effect correction is presented in **Figure 3**. Before the application of *ComBat*, most variation is observed between the two different datasets (**Figures 3A, C**), while after the application of *ComBat*, most variation is observed between the different class labels (**Figures 3B, D**). Therefore, *ComBat* successfully removed the batch effect between the two datasets.

### Rejection Classifier Development

#### B-HOT Model

The expression values of the 18,945 genes that remained after probe-set comparison were filtered in order to create a feature set containing only the genes included in the B-HOT panel. The genes CMV *UL83*, BK *TAg*, BK *VP1*, and EBV *LMP2* are all virus-related biomarkers of tissue damage that are therefore not included in the Affymetrix Human Genome arrays. Despite their potential value inside the B-HOT panel for what concerns other Banff diagnostic categories, they should not be informative for what concerns ABMR and TCMR diagnosis. Four other genes (*OR2I1P, MT1A, MIR155HG*, and *IGHG4*) from the B-HOT panel were missing in at least one of the two microarray versions and consequently absent in the resulting preprocessed training set. Therefore, the final training set based on the GSE98320 dataset includes 762 genes for 1,181 samples.

**Figure 4:**
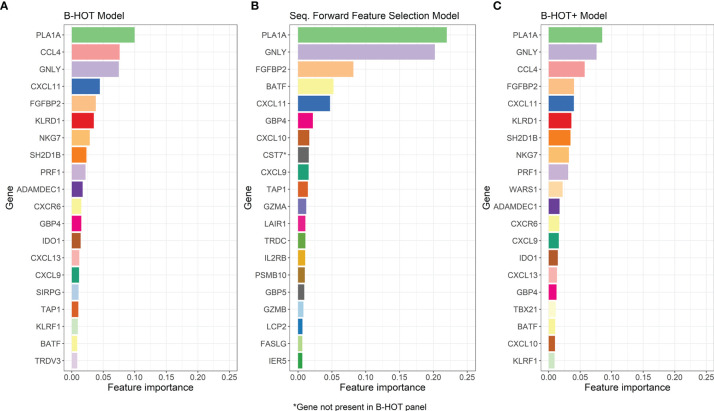
Overview of the Most-Predictive Features of the three Random Forest Models. **(A)** The twenty most predictive features for classification within GSE98320 of the B-HOT Model. **(B)** The twenty most predictive features for classification within GSE98320 of the Forward Sequential Feature Selected Model. **(C)** The twenty most predictive features for classification within GSE98320 of B-HOT+ Model. B-HOT, Banff-Human Organ Transplant.

Precision/Recall and receiver operating characteristic (ROC) curves with corresponding AUCs of the developed model are presented in **Figure S1**. During cross-validation, the model showed an average overall accuracy of 0.913. Additional performance scores are displayed in **Table 3** and **Table S1**. The most predictive features of this model are displayed in **Figure 4A**. The list of all genes with corresponding importance is provided in **Table S2A**.

**Table 3:** Overview of nested cross-validation performances of all models.

Model	Performance	NR	ABMR	TCMR	
**B-HOT Model**	AUC	0.980	0.976	0.995	
	Precision	0.924	0.893	0.900	
	Recall	0.960	0.835	0.779	
	F1-score	0.941	0.861	0.827	
	Average Accuracy				0.913
**Feature Selection Model**	AUC	0.979	0.971	0.987	
	Precision	0.926	0.891	0.813	
	Recall	0.963	0.825	0.728	
	F1-score	0.944	0.855	0.759	
	Average Accuracy				0.909
**B-HOT+ Model**	AUC	0.980	0.976	0.994	
	Precision	0.936	0.886	0.921	
	Recall	0.963	0.862	0.767	
	F1-score	0.949	0.874	0.828	
	Average Accuracy				0.921

ABMR, Antibody-Mediated Rejection; AUC, Area under the ROC curve; B-HOT, Banff-Human Organ Transplant; NR, Non-Rejection; TCMR, T-Cell-Mediated Rejection.

#### Feature Selection Model

To investigate whether a model originating from feature selection applied to the complete training set performs better than one based on the B-HOT panel and to find additional predictive features, a second random forest model was developed. This model was based on sequential feature selection using k-nearest neighbors to identify the one hundred most predictive genes out of the 18,945 genes overlapping between the training and validation datasets.

Precision/Recall and ROC curves with corresponding AUCs are presented in **Figure S2**. During cross-validation, the model showed an average cross-validation accuracy of 0.909. Additional performance scores are presented in **Table 3** and **Table S1**. The most predictive features of this model are displayed in **Figure 4B**. The list of the 100 selected features is provided in **Table S2B**.

The performances of the second model were generally lower compared to the first B-HOT-based one.

#### B-HOT+ Model

To assess if other genes could have added value to the B-HOT model to improve classification performances, a third model was developed based on the B-HOT panel with the addition of the most predictive genes from the Feature Selection model that are not included in the B-HOT panel itself. Sequential addition of these genes ranked according to their importance was performed until the next gene would not improve model performances. Six genes (*CST7, KLRC4-KLRK1, TRBC1, TRBV6-5, TRBV19*, and *ZFX*) were added to the feature set. The final training set based on the GSE98320 dataset, therefore, included 768 genes for 1,181 samples.

**Figure 5:**
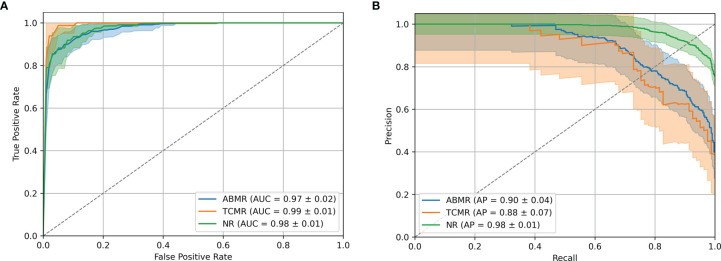
B-HOT+ Model Performances. **(A)** ROC curve of cross-validation within GSE98320 of the B-HOT+ model. **(B)** Precision/Recall curve of cross-validation within GSE98320 of the B-HOT+ model. ABMR, Antibody-Mediated Rejection; AP, Average Precision; AUC, Area Under the ROC Curve; B-HOT, Banff-Human Organ Transplant; NR, Non-Rejection; ROC, Receiver Operating Characteristic; TCMR, T-Cell-Mediated Rejection.

Precision/Recall and ROC curves with corresponding AUCs are presented in **Figure 5**. During cross-validation, the model showed an average accuracy of 0.921. Additional performance scores are displayed in **Table 3** and **Table S1**. The most predictive features of this model are displayed in **Figure 4C**. The list of all genes with corresponding importance is provided in **Table S2C**.

The performance scores of the third model were generally higher compared to the others and therefore the B-HOT+ model was chosen as the best performing classifier to validate on an external dataset.

### Measuring Performance Using Independent Micro-Array Data

As previously mentioned, the selected external validation set GSE129166 is composed of 77 samples (60 NR, 15 ABMR, and 2 TCMR). Classification of the 77 samples using our third B-HOT+ model was performed.

**Figure 6:**
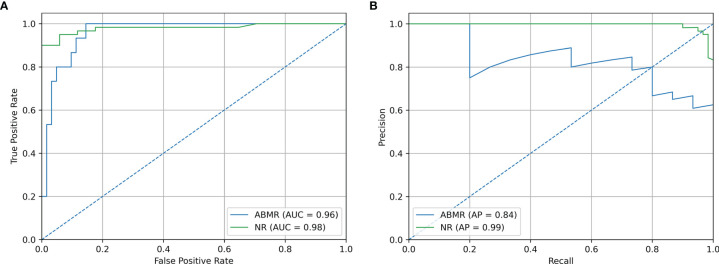
Validation Set Performances. **(A)** ROC curve of independent validation within GSE129166 of the B-HOT+ model. **(B)** Precision/Recall curve of independent validation within GSE129166 of the B-HOT+ model. ABMR, Antibody-Mediated Rejection; AP, Average Precision; AUC, Area Under the ROC Curve; B-HOT, Banff-Human Organ Transplant; NR, Non-Rejection; ROC, Receiver operating characteristic.

Precision/Recall and ROC curve with corresponding AUCs are presented in **Figure 6**. On this external dataset, the model showed an accuracy of 0.883, and an AUC of 0.965 and 0.982 for NR and ABMR respectively. Performances concerning the TCMR class are not reported considering the insufficient sample size, namely 2 samples. This limitation is addressed in the Discussion section. Additional performance scores are displayed in **Table 4**. A confusion matrix reporting on the prediction performance of this model is reported in **Table S3**.

**Table 4:** Overview of B-HOT+ Model Validation Performances.

Model	Performance	NR	ABMR	TCMR
**B-HOT+ Model Validation**	AUC	0.965	0.982	X^*^
	Precision	0.65	1.000	X^*^
	Recall	0.867	0.862	X^*^
	F1-score	0.742	0.938	X^*^

*Insufficient sample size.

ABMR, Antibody-Mediated Rejection; AUC, Area under the ROC curve; B-HOT, Banff-Human Organ Transplant; NR, Non-Rejection; TCMR, T-Cell-Mediated Rejection.

## Discussion

In this study, we developed a decentralized molecular kidney transplant biopsy classifier to improve the diagnosis of transplant rejection and subsequently improve the classification of the type of rejection, namely ABMR and TCMR. Moreover, we compared the discriminating power of the B-HOT panel to that of forward sequential feature selection applied to the whole microarray gene set and found 6 additional predictive genes that increase the B-HOT panel performances in a clinical scenario. The resulting B-HOT+ model achieved an average accuracy of 0.921 during cross-validation and AUCs of 0.965 and 0.982 for NR and ABMR respectively on an external validation set.

To our knowledge, we developed the first kidney transplant biopsy classifier that is able to classify NR and ABMR samples within a validation set derived from a different analysis platform, in this case, a different microarray version. This is a large step towards the development of a thoroughly validated and multi-platform compatible model that will require a coordinated and organic effort from multiple members of the research community. The MMDx is the main currently available molecular diagnostic system for transplant rejection. Its strength relies on its thoroughly validated gene-set and centralized analysis pipeline. This system strongly advanced research in the seek for a reliable molecular diagnostic system for allograft pathology, but its centralized nature limits its availability and clinical impact. Further research is needed for the development of a decentralized molecular diagnostic system for allograft pathology and general agreements over specific methodologies, such as predefined gene-sets or analysis pipelines, might be of benefit to produce more coherent and comparable literature, speeding up the development process.

In our study, the B-HOT panel appeared to be a high-quality and reliable gene-set for the classification of kidney transplant biopsies, supporting the findings of other research groups investigating the discriminatory value of this knowledge-based panel for kidney transplant diagnostics (15). An alternative model built with feature selection performed over the whole gene-set failed to outperform the B-HOT panel-based model. The superior performances of the B-HOT panel compared to this technique reinforce our will to support and implement its use. We consider the B-HOT panel an important milestone of the Banff Foundation and the whole transplantation community, and further studies are needed to optimize its composition and clinical impact.

The six additional genes (*CST7, KLRC4-KLRK1, TRBC1, TRBV6-5, TRBV19*, and *ZFX)* derived from the Feature Selection model improved the predictive power of the B-HOT panel. These genes appeared to be involved in the regulation and action of the immune function, suggesting that their additional predictive power is unlikely to be due to chance. *CST7* and *ZFX* act on hematopoietic cell precursors, regulating the immune function (16, 17). *KLRC4-KLRK1* takes part in the innate immune function and regulates natural killer cells (18). *TRBC1*, *TRBV6-5*, and *TRBV19* are all genes coding for T cell receptor structures (19). All things considered, performances improved when these genes were added to the current B-HOT panel, suggesting that they should be further investigated and possibly considered for the optimization of the B-HOT panel itself.

This study has several limitations. First of all, the retrospective nature of this study limits the strength of its findings. Furthermore, the GSE98320 training set is composed of 1,181 biopsy samples with corresponding expression levels and MMDx archetype diagnoses. However, our validation set GSE129166 reports histological diagnoses (Banff diagnostic categories), displaying the validity of the MMDx archetypes and again raising the question about what we should consider as ground truth considering the high interobserver variability that characterizes histological renal allograft diagnoses. Finally, our validation set presents only 2 TCMR samples, preventing us from evaluating our model’s performances concerning that diagnostic category. However, gene expression analysis support for ABMR diagnosis has been long-awaited by the community, considering the drawbacks and complexities of ABMR histopathological diagnosis and subtyping. For this reason, our main focus was on the validation of performances for the diagnostic category of ABMR.

The development of the commercially available B-HOT panel is a large achievement of the Banff Foundation for Allograft Pathology in collaboration with NanoString^®^. Subsequently, the integration of a multi-platform compatible diagnostic system to diagnose kidney allograft pathology with this technology should be a priority. Multi-platform compatibility is vital as single-platform models could limit the availability of newly developed diagnostic tools. Although the price for a NanoString^®^ sample run is lower than the ones of many other gene expression analysis technologies, the use of NanoString^®^ could be demanding for what concerns set-up and use. RNA in formalin-fixed paraffin-embedded biopsies is prone to degradation. Therefore, the NanoString^®^ technology is very suitable for this type of sample and can drastically increase the possibility of obtaining large datasets. Furthermore, gene expression data from different technologies already showed to be highly correlated (20, 21). A multi-platform compatible system will allow different centers to use already available gene expression analysis systems. For this to be possible, specific normalization pipelines for different gene analysis technologies are vital to combine different datasets, virtually expanding the available data for the development of the diagnostic system itself.

In conclusion, we developed a molecular diagnostic model for renal allograft pathology that is able to work on different microarray versions, taking a large step in the final development of a decentralized multi-platform compatible system that could strongly influence clinical practice and outcome. Further research is needed, especially focusing on the complex normalization pipelines that are required to compare gene expression data generated by different technologies. The development of this tool must combine the efforts of the whole transplantation community for the validation of the B-HOT panel, making sure that optimal performances are achieved through the use of this technology. The current B-HOT panel proved to be an extensive and reliable gene-set for kidney transplant biopsy classification, however, we found that the addition of 6 genes could lead to a superior B-HOT+ model to classify transplant rejection.

## Data Availability Statement

Publicly available datasets were analyzed in this study. This data can be found here: https://www.ncbi.nlm.nih.gov/geo/, GSE98320, GSE129166.

## Author Contributions

MB and IC have contributed equally to this work and share first authorship. MB, IC, MC, RM, AS, JJ, and YL contributed to the conception and design of the study. MB developed the classifiers with the support of IC. MB and IC drafted the manuscript. JJ reviewed the classifier script. MC, RM, AS, YL, MR, JJ, and HV reviewed the manuscript. All authors contributed to the article and approved the submitted version.

## Conflict of Interest

Author MB was employed by company Omnigen BV.

The remaining authors declare that the research was conducted in the absence of any commercial or financial relationships that could be construed as a potential conflict of interest.

## Publisher’s Note

All claims expressed in this article are solely those of the authors and do not necessarily represent those of their affiliated organizations, or those of the publisher, the editors and the reviewers. Any product that may be evaluated in this article, or claim that may be made by its manufacturer, is not guaranteed or endorsed by the publisher.

## Abbreviations

ABMR, Antibody-mediated rejection; AUC, Area under the ROC curve; B-HOT, Banff-Human Organ Transplant; CV, Cross-validation; MMDx, Molecular Microscope^®^ Diagnostic System; NR, Non-rejection; ROC, Receiver operating characteristic; TCMR, T-cell-mediated rejection.
