# Supplementary figures and images for: A Decentralized Kidney Transplant Biopsy Classifier for Transplant Rejection Developed Using Genes of the Banff-Human Organ Transplant Panel
Source: Front Immunol. 2022 May 10;13:841519. doi: 10.3389/fimmu.2022.841519 (PMC9128066; doi:10.3389/fimmu.2022.841519)

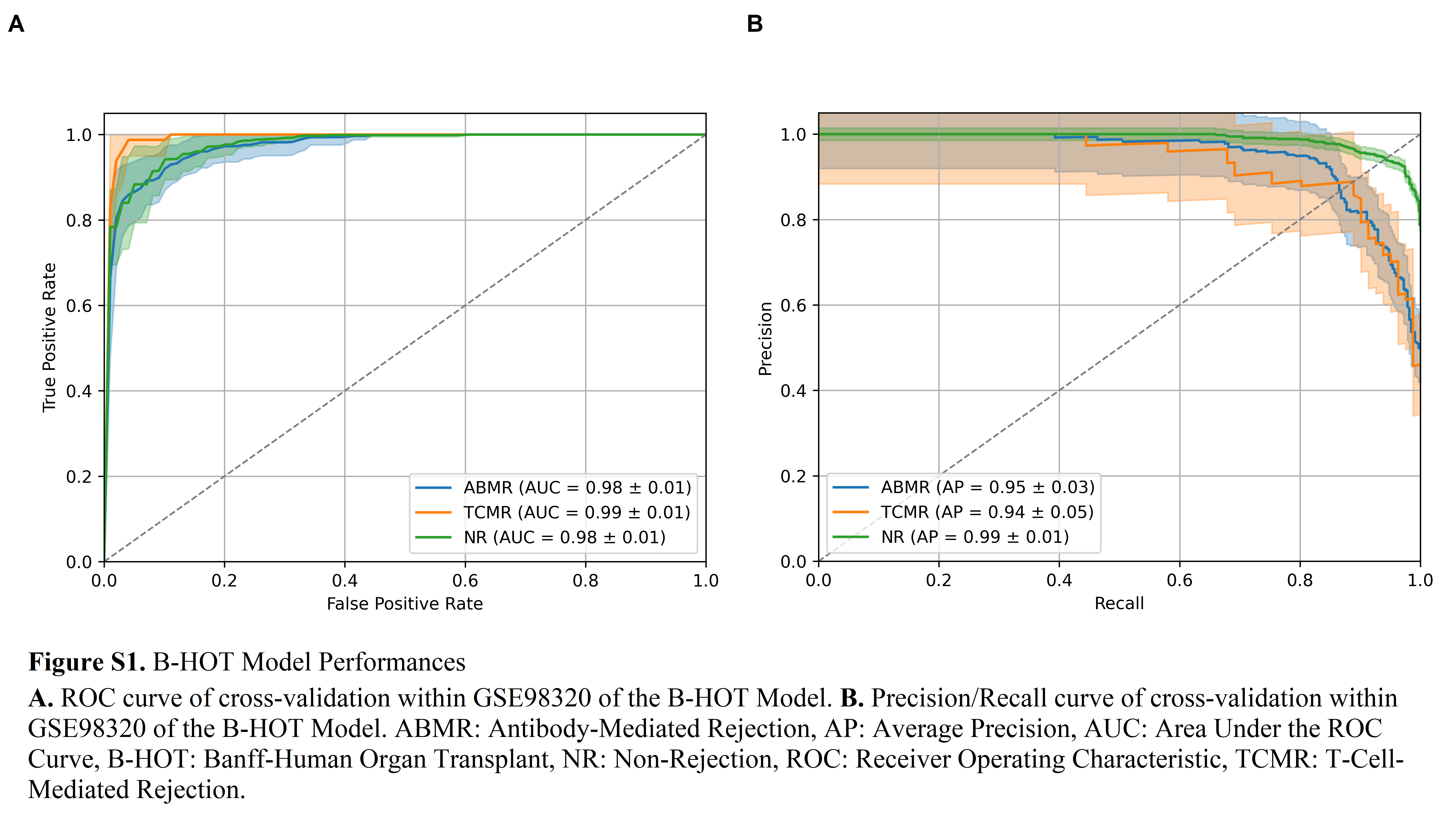

Supplement: Supplementary file 1 [file Image_1.tiff]

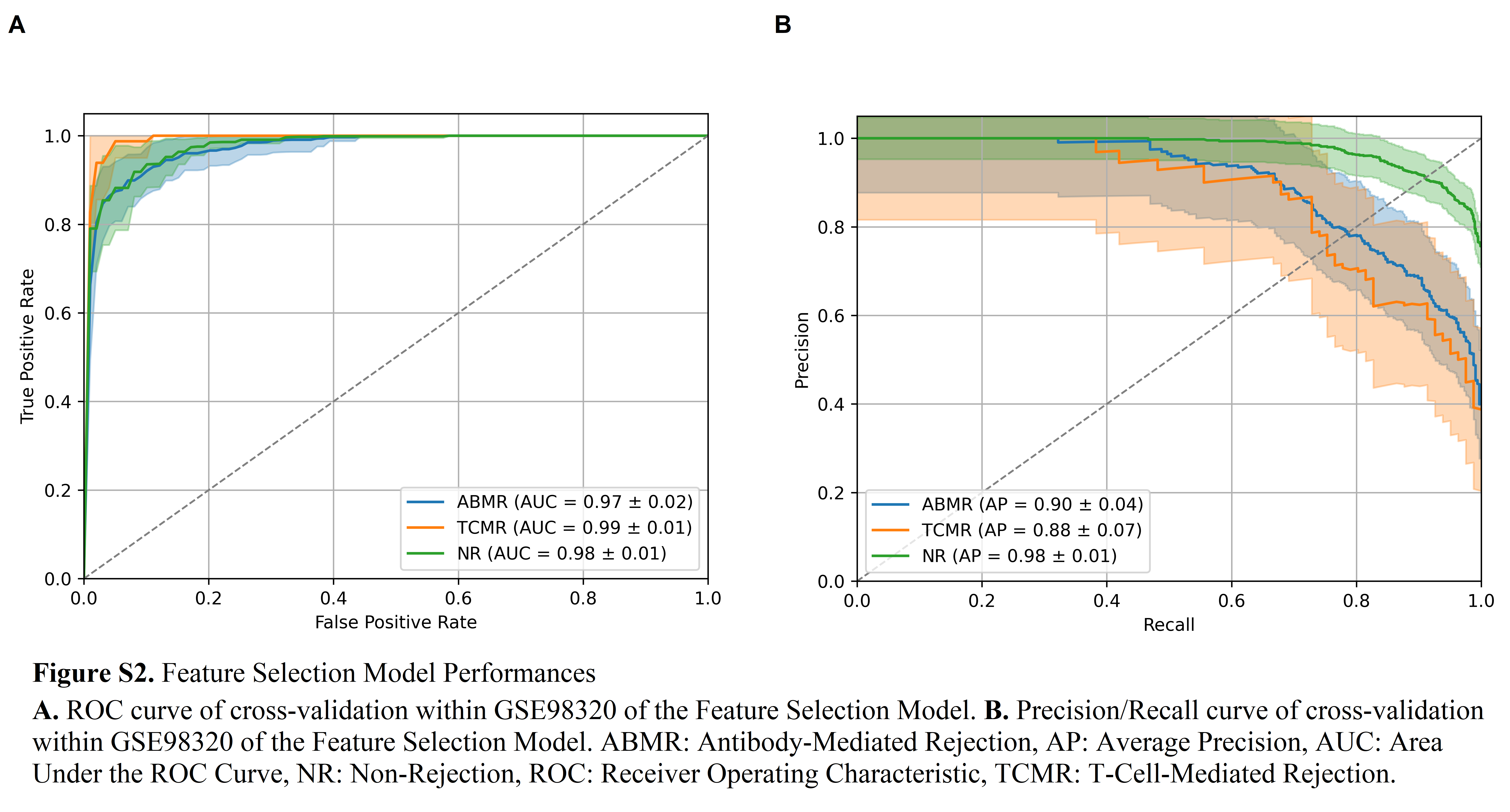

Supplement: Supplementary file 2 [file Image_2.tiff]
