# Supplementary material for: A Decentralized Kidney Transplant Biopsy Classifier for Transplant Rejection Developed Using Genes of the Banff-Human Organ Transplant Panel
Source: Front Immunol. 2022 May 10;13:841519. doi: 10.3389/fimmu.2022.841519 (PMC9128066; doi:10.3389/fimmu.2022.841519)
Supplement: Supplementary file 5 [file Table_3.docx]

**Table S3.** B-HOT+ Model Validation Confusion Matrix

ABMR: Antibody-Mediated Rejection, B-HOT, Banff-Human Organ Transplant, NR: Non-Rejection, TCMR: T-Cell-Mediated Rejection.

|  | | Predicted labels | | |
| --- | --- | --- | --- | --- |
|  |  | ABMR | TCMR | NR |
| True labels | ABMR | 13 | 2 | 0 |
|  | TCMR | 0 | 2 | 0 |
|  | NR | 7 | 0 | 53 |
